# Finerenone is associated with pronounced uric acid reduction in hyperuricemic diabetic kidney disease: a real-world analysis

**DOI:** 10.3389/fphar.2026.1782658

**Published:** 2026-03-02

**Authors:** Yanmei Lin, Jianqing Tian, Kang Du, Bo Liu

**Affiliations:** 1 Xiamen Humanity Hospital, Xiamen, Fujian, China; 2 Southern Business Group, ZoeSoft Company Limited, Xiamen, Fujian, China; 3 The First Affiliated Hospital of Xiamen University, Xiamen, Fujian, China

**Keywords:** diabetic kidney disease, finerenone, hyperuricemia, mineralocorticoid receptor antagonist, uric acid

## Abstract

**Background:**

Diabetic kidney disease (DKD) is a critical complication of type 2 diabetes, often compounded by hyperuricemia, which may accelerate renal function decline. While finerenone, a nonsteroidal mineralocorticoid receptor antagonist (MRA), provides renal and cardiovascular benefits, its impact on uric acid (UA) metabolism in real-world DKD patients, particularly those with high baseline SUA, remains controversial.

**Methods:**

In this retrospective, single-center study, we included 124 patients with type 2 DKD (baseline eGFR ≥60 mL/min/1.73 m^2^) who initiated finerenone. Patients with recent gout or urate-lowering therapy were excluded. Changes in SUA, urinary albumin-to-creatinine ratio (UACR), and eGFR were assessed before and after 1–3 months of treatment. Statistical analyses employed linear mixed models for longitudinal data and multivariable regression.

**Results:**

Linear mixed model analysis showed finerenone treatment was associated with a significant reduction in SUA (adjusted mean difference: −47.9 μmol/L, 95% CI: −63.5 to −32.3; p < 0.001). This reduction was substantially greater in patients with baseline hyperuricemia (−88.6 μmol/L) than in those without (−16.6 μmol/L; p for interaction = 0.003). UACR decreased by 39.4% (p < 0.001), while eGFR showed a small but significant decline (−2.7 mL/min/1.73 m^2^, p = 0.019). The SUA-lowering association was independent of concomitant SGLT2 inhibitor or GLP-1 receptor agonist use in multivariable analyses. Hyperkalemia (potassium ≥5.5 mmol/L) occurred in 0.8% of patients.

**Conclusion:**

In this real-world cohort, finerenone use was associated with significant reductions in albuminuria and SUA, particularly among patients with hyperuricemia. These findings suggest a potential dual benefit in this high-risk subgroup and highlight the importance of baseline SUA in interpreting finerenone’s metabolic effects. The observed SUA reduction warrants further prospective investigation.

## 1 Introduction

Diabetic kidney disease (DKD), a major microvascular complication of diabetes, is a leading cause of end-stage renal disease (ESRD) and significantly elevates cardiovascular risk (Agarwal et al., 2022). Current management strategies focus on glycemic, blood pressure, and lipid control, alongside renin-angiotensin system (RAS) blockade (Alicic and Neumiller, 2025; Alhomoud et al., 2025). Despite these interventions, a substantial residual risk of renal function decline persists, underscoring the urgent need for novel therapeutic avenues.

Mineralocorticoid receptor (MR) overactivation is a well-established central driver of inflammation and fibrosis in DKD pathogenesis (Alhomoud et al., 2025). Concurrently, hyperuricemia has emerged as an independent risk factor for renal injury and DKD progression (American Diabetes Association, 2025). Elevated uric acid (UA) levels contribute to renal damage through oxidative stress, intrarenal RAS activation, and pro-inflammatory pathways (Bakris et al., 2020). Furthermore, a vicious cycle ensues: DKD impairs UA excretion, and resultant hyperuricemia exacerbates renal injury, accelerating disease progression (Benvenuto et al., 2025).

Finerenone, a third-generation nonsteroidal MR antagonist (MRA), has demonstrated significant cardiorenal benefits in pivotal clinical trials (FIDELIO-DKD and FIGARO-DKD), positioning it as a cornerstone therapy for DKD (Calcagno et al., 2025; Chinese Expert Consensus on Finerenone Clinical Application Writing Group, 2024). However, its impact on UA metabolism remains contentious. While landmark trials reported neutral or slight increases in serum UA (SUA), small-scale observational studies have yielded conflicting results (Chinese FIDELITY Study Group, 2023; Chinese Guidelines for the Diagnosis, 2024). These discrepancies likely stem from methodological limitations, such as unstratified patient cohorts with varying baseline SUA levels and inadequate exclusion of acute gout flares or concomitant urate-lowering therapies—key confounders in assessing UA dynamics.

To address these gaps, this retrospective study evaluated short-term (1–3 months) changes in SUA following finerenone initiation in a real-world cohort of 124 patients with proteinuric DKD and preserved renal function, who were free of recent gout. By employing stricter patient selection and accounting for prior methodological shortcomings, this study aims to clarify the association between finerenone treatment and SUA changes, particularly in individuals with baseline hyperuricemia. Elucidating this relationship may reveal an additional metabolic dimension to finerenone’s therapeutic profile and inform its optimized use in high-risk DKD subpopulations.

## 2 Materials and methods

### 2.1 Patients and Procedures

This retrospective cohort study included patients with type 2 diabetic kidney disease (T2DKD) treated with finerenone at Xiamen Humanity Hospital Endocrinology Department (January 2023–May 2025). Diagnosis followed 2025 ADA criteria: urinary albumin-to-creatinine ratio (UACR) ≥30 mg/g or 24 h urinary albumin ≥30 mg or eGFR <60 mL/min/1.73 m^2^ sustained ≥3 months after excluding other causes (Epstein, 2008).

Inclusion required: ADA-defined T2DKD; baseline eGFR ≥60 mL/min/1.73 m^2^; finerenone treatment ≥1 month (stable dose 10/20 mg/day adjusted by eGFR); complete pre-treatment (≤1 month) and post-treatment (1–3 months) lab records.

Exclusion criteria: active/treated malignancy (≤1 year); rheumatic disease (e.g., SLE, RA); acute gout/uric acid-lowering drug use (≤3 months); Child-Pugh ≥ B liver dysfunction; pregnancy/lactation; baseline serum potassium ≥5.0 mmol/L or finerenone contraindications; potassium/uric acid-modifying drug use (≤3 months).

The patient selection process is summarized in Supplementary Figure S1.

### 2.2 Data collection

Baseline data were collected within 1 month prior to finerenone initiation, including demographic characteristics (age, sex), metabolic profiles (fasting plasma glucose, HbA1c, triglycerides, serum uric acid, urine pH), renal parameters (urine albumin-to-creatinine ratio, serum creatinine, estimated glomerular filtration rate calculated via CKD-EPI equation), serum potassium levels, and concurrent medication use (GLP-1 receptor agonists, SGLT2 inhibitors, and initial finerenone dose of 10 mg/day). Follow-up assessments at 1–3 months post-treatment involved repeated measurements of all baseline parameters, with specific focus on changes in urine albumin-to-creatinine ratio, estimated glomerular filtration rate, and serum uric acid levels, as well as monitoring for treatment-emergent hyperkalemia (defined as serum potassium ≥5.5 mmol/L). All laboratory tests were conducted under standardized fasting conditions.

### 2.3 Definitions and assignment

Hyperuricemia was defined as a baseline SUA level >420 μmol/L for men or >360 μmol/L for women.

The primary endpoint was the absolute change in SUA from baseline to follow-up. Subgroup analyses compared this change between patients with and without baseline hyperuricemia. Secondary endpoints included: (1) the relative change in UACR; and (2) the absolute change in eGFR (Fidelity Study Group, 2024a; Fidelity Study Group, 2024b).

Safety assessments focused on the incidence of treatment-emergent hyperkalemia (defined as serum potassium ≥5.5 mmol/L on at least one consecutive measurement after treatment initiation).

The finerenone dosing strategy adhered to the prescribing information: an initial dose of 10 mg once daily for patients with serum potassium ≤5.0 mmol/L and eGFR ≥25 mL/min/1.73 m^2^. The dose could be escalated to 20 mg once daily after 4 weeks if serum potassium was ≤4.8 mmol/L and eGFR had not declined by >30% from baseline. The dose was maintained at 10 mg if serum potassium was between 4.8 and 5.5 mmol/L or if eGFR decline exceeded 30%. Finerenone was discontinued if serum potassium exceeded 5.5 mmol/L and could be re-initiated at 10 mg once daily once it fell to ≤5.0 mmol/L (Halimi et al., 2025).

## 3 Statistical analysis

Data analysis was conducted using R software. Continuous variables are presented as mean ± SD or median (IQR), and categorical variables as counts (%). Normality was assessed using the Shapiro-Wilk test and Q-Q plots. Changes in SUA, UACR, and eGFR were analyzed with linear mixed models (time as fixed effect, patient random intercept), with results reported as model-estimated mean differences and 95% CIs. Between-group comparisons used t-tests or Mann-Whitney U tests. Predictors of SUA reduction were identified via LASSO regression (10-fold CV, optimal λ by minimum MSE), followed by multivariable regression. Given the exploratory nature, results focus on association direction and strength. P < 0.05 was considered significant.

## 4 Results

### 4.1 Patient baseline characteristics

A total of 124 patients diagnosed with type 2 diabetic kidney disease (DKD) were included in this retrospective analysis. The baseline demographic, clinical, and laboratory characteristics are summarized in Table 1. The mean age was 55.8 ± 11.6 years, with males comprising 64.5% of the cohort. A significant proportion of patients were on concurrent medications, including SGLT2 inhibitors (65.3%) and GLP-1 receptor agonists (38.7%). At baseline, 44.4% (n = 55) of patients met the criteria for hyperuricemia.

**TABLE 1:** Baseline characteristics of study population.

Category	Variables	Total (n = 124)
Demographics		
​	Age, mean ± SD (years)	55.82 ± 11.55
​	Sex female, n (%)	44 (35.48)
​	BMI, M (Q_1_, Q_3_) (kg/m^2^)	24.93 (22.50, 27.18)
Comorbidities		
​	Hypertension, n (%)	69 (55.65)
Laboratory parameters		
​	Scr, mean ± SD (μmol/L)	74.65 ± 21.16
​	FFP, mean ± SD	7.31 ± 1.40
​	Baseline UACR (mg/g), mean ± SD	376.57 ± 529.66
​	Baseline SUA (μmol/L), mean ± SD	400.0 ± 89.1
​	Baseline eGFR (mL/min/1.73 m^2^), mean ± SD	91.44 ± 20.47
​	HbA1c, M (Q_1_, Q_3_) (%)	7.95 (6.80, 9.62)
​	TG, M (Q_1_, Q_3_) (mmol/L)	2.05 (1.17, 3.31)
​	Urine pH, M (Q_1_, Q_3_)	5.50 (5.50, 6.00)
Medications		
​	GLP-1 agonist users, n (%)	48 (38.71)
​	SGLT2 inhibitor users, n (%)	81 (65.32)
​	Finerenone dose, n (%)	​
​	−10 mg	48 (38.71)
​	−20 mg	76 (61.29)

FFP, fasting plasma glucose.

### 4.2 Changes in key outcomes evaluated by linear mixed model

**FIGURE 1:**
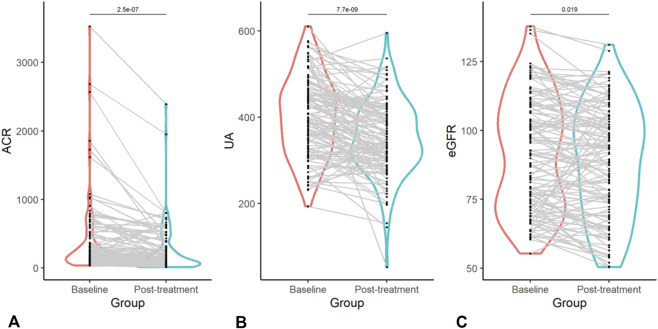
Comparative analysis of key clinical parameters before and after finerenone administration. Note: This figure consists of three panels, each presenting the changes in a specific clinical parameter before (Baseline) and after (Post-treatment) finerenone administration in patients with type 2 diabetic kidney disease (T2DKD). **(A)** (Albumin-to-Creatinine Ratio, ACR): Illustrates the reduction in urinary albumin excretion, a marker of renal injury. **(B)** (Serum Uric Acid, UA): Demonstrates the decline in serum uric acid levels, highlighting the potential metabolic effects of finerenone. **(C)** (Estimated Glomerular Filtration Rate, eGFR): Shows the slight but statistically significant decrease in eGFR, consistent with the acute hemodynamic effects of mineralocorticoid receptor antagonists (MRAs). All parameters were measured under standardized fasting conditions, and statistical comparisons were performed using linear mixed models (LMMs) to account for within-patient correlations.

The impact of finerenone on key clinical parameters was assessed using a linear mixed model (LMM) for repeated measures data, with results presented in Table 4. Serum Uric Acid (SUA): LMM analysis demonstrated a significant reduction in SUA levels after treatment, with an adjusted mean difference of −47.9 μmol/L (95% CI: −63.5 to −32.3; p < 0.001). Urinary Albumin-to-Creatinine Ratio (UACR): UACR decreased significantly, with a mean reduction of −148.2 mg/g (95% CI: −202.6 to −93.9; p < 0.001),corresponding to a 39.4% decline from baseline. Estimated Glomerular Filtration Rate (eGFR): A small but statistically significant decline in eGFR was observed (mean change: −2.7 mL/min/1.73 m^2^; 95% CI: −5.0 to −0.4; p = 0.019). The individual trajectories and paired changes for UACR, SUA, and eGFR in each patient are visually presented in Figure 1. Paired t-test analysis further verified the significant changes in these key parameters, with detailed results shown in Table 2.

**TABLE 2:** Paired t-test Results (Baseline vs. Post-treatment).

Variables	Mean difference (baseline - post) ± SD	95% CI of difference	t-value	df	P-value
UACR (mg/g)	148.25 ± 303.92	93.9–202.6	5.426	123	<0.001
SUA (μmol/L)	47.9 ± 86.8	32.3–63.5	6.256	123	<0.001
eGFR (mL/min/1.73 m^2^)	2.72 ± 12.69	0.42–5.02	2.386	123	0.019

The Mean Difference for eGFR, is positive here as it represents Baseline - Post, indicating an overall decline in eGFR, post-treatment.

### 4.3 Subgroup and interaction analyses

The reduction in SUA was heterogeneous across predefined subgroups (Table 3). Hyperuricemia Status: The SUA-lowering effect was markedly more pronounced in patients with baseline hyperuricemia (−88.6 μmol/L; 95% CI: −104.7 to −72.5) compared to those without (−16.6 μmol/L; 95% CI: −29.8 to −3.4), with a statistically significant interaction (p for interaction = 0.003). Concomitant Medications: The magnitude of SUA reduction was not significantly modified by concomitant use of SGLT2 inhibitors (Heerspink et al., 2019) (p for interaction = 0.750) or GLP-1 receptor agonists (p for interaction = 0.680).

**TABLE 3:** Linear mixed model analysis of key outcomes before and after treatment.

Parameter	Baseline estimate ± SE	Post-treatment estimate ± SE	Adjusted mean difference (95% CI)	P-value	Subgroup analysis (mean difference, 95% CI)	Interaction P-value
Serum uric acid (μmol/L)	400.0 ± 8.02	352.1 ± 7.76	−47.9 (−63.5, −32.3)	<0.001	Hyperuricemia (n = 55): −88.6 (−104.7, −72.5) SGLT2i Users (n = 81): −46.8 (−62.9, −30.7) GLP-1 users (n = 48): −45.2 (−66.5, −23.9)	Hyperuricemia: 0.003 SGLT2i: 0.750 GLP-1: 0.680
UACR (mg/g)	376.57 ± 47.79	228.32 ± 29.71	−148.2 (−202.6, −93.9)	<0.001	-	-
eGFR (mL/min/1.73 m^2^)	91.44 ± 2.56	88.72 ± 2.53	−2.7 (−5.0, −0.4)	0.019	-	-

^a^ Reference group for categorical variables.

^b^ β represents the standardized regression coefficient.

^c^ Model adjusted for all variables listed in the table.

^d^ Statistical significance at P < 0.05 level.

### 4.4 Predictors of SUA reduction

**FIGURE 2:**
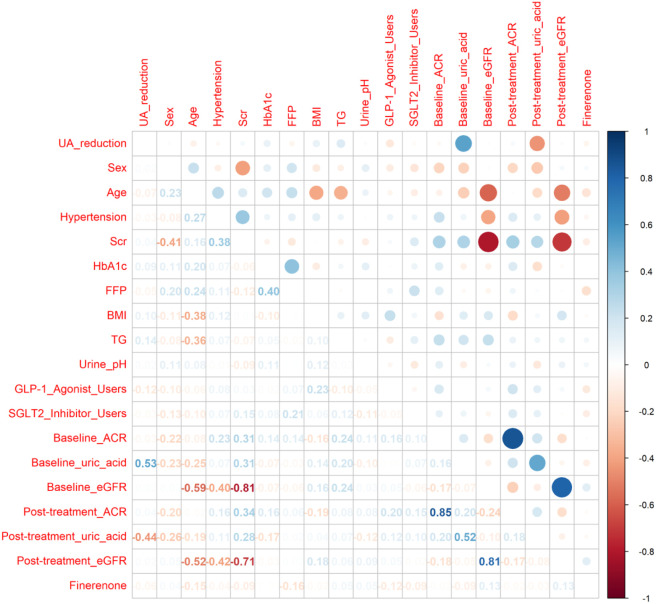
Pearson correlation coefficients between UA reduction and clinical variables. Note:This figure displays the Pearson correlation coefficients (r) between the magnitude of serum uric acid (UA) reduction (ΔSUA) and multiple baseline clinical variables. The heatmap-style presentation allows for visual identification of variables with stronger (red) or weaker (blue) associations with UA reduction. Key variables include:HbA1c: Glycated hemoglobin, reflecting long-term glycemic control. Baseline SUA: Initial serum uric acid level, a critical predictor of UA reduction. eGFR: Estimated glomerular filtration rate, indicating renal function. Triglycerides (TG): Lipid profile component, potentially linked to metabolic syndrome. Correlation coefficients were derived from unadjusted bivariate analyses, and statistical significance was set at P < 0.05.

The unadjusted correlations between the magnitude of SUA reduction (ΔSUA) and other continuous clinical variables are displayed in Figure 2.

**FIGURE 3:**
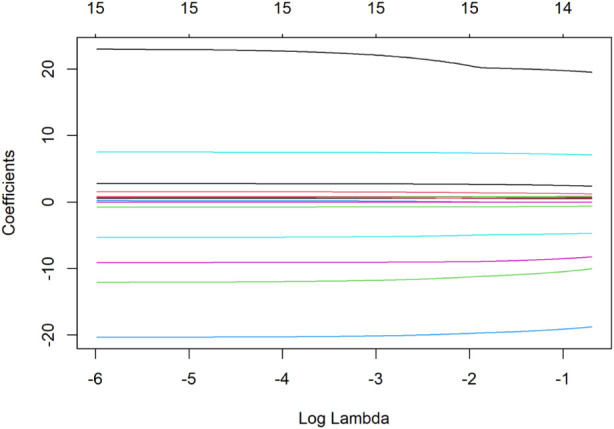
Lasso regression for predictor screening. Note:Ten-fold cross-validation is employed for Lasso regression, and the value of lambdas corresponding to the minimum mean squared error is selected as the optimal solution. X-axis (Lambda, λ): Represents the penalty strength in Lasso regression. As λ increases, fewer variables are retained in the model. Y-axis (Coefficients): Shows the standardized regression coefficients for each variable. Variables with non-zero coefficients at the optimal λ (minimum mean squared error, MSE) were selected for multivariable analysis. Optimal λ: Marked by the vertical dashed line, corresponding to the λ value with the lowest cross-validated MSE. Key predictors retained in the model include baseline SUA, HbA1c, and fasting blood glucose.

**FIGURE 4:**
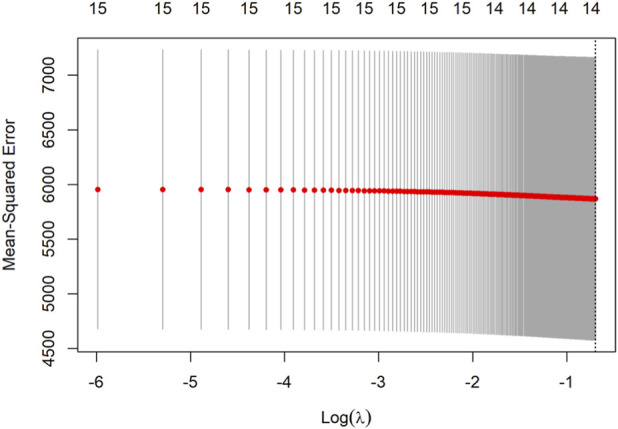
Multivariable linear regression results for UA reduction predictors. Note:This figure presents the results of multivariable linear regression analysis, identifying independent predictors of serum uric acid (UA) reduction (ΔSUA) after finerenone treatment.X-axis (Predictors): Lists the variables included in the final model, selected via Lasso regression. Y-axis (Regression Coefficients, β): Represents the magnitude and direction of the association between each predictor and ΔSUA. Error Bars: Denote the 95% confidence intervals (CIs) for each β estimate.

To identify independent predictors, candidate variables were first screened using LASSO regression with 10-fold cross-validation (Figure 3), where the optimal lambda (λ) was selected at the point of minimum mean squared error. The regression coefficients of all variables screened by LASSO regression are presented in Table 4, and the optimal lambda (λ) was selected at the point of minimum mean squared error. This process identified 14 candidate variables for inclusion in the final multivariable linear regression model (results detailed in Table 5 and visualized in Figure 4).

**TABLE 4:** LASSO regression coefficients for UA reduction predictors (S1).

Variable	S1
Sex	19.52217
Age	1.223,046
Hypertension	−10.03895
Glycated hemoglobin	7.075977
FBG	−8.242,461
BMI	2.393,835
TG	0.7174784
Urine pH	0.8895219
GLP_1	−18.76458
SGLT2	−4.692,044
Baseline ACR	−0.0005136087
Baseline uric acid	0.5674979
GFR	0.4537650
Finerenone	−0.6209937

During Lasso regression, ten-fold cross-validation is used, and the value of lambdas corresponding to the minimum mean squared error is selected as the optimal solution.

**TABLE 5:** Multivariable linear regression analysis for UA reduction predictors.

Variable	β	S.E	t	β (95% CI)	P
Sex	20.47	15.28	1.34	−9.47–50.42	0.183
Age	1.46	0.96	1.51	−0.43–3.34	0.133
Hypertension	−11.66	15.69	−0.74	−42.42–19.10	0.459
HbA1c (%)	7.48	3.59	2.08	0.44–14.51	0.040
Fasting blood glucose	−9.25	5.74	−1.61	−20.51–2.00	0.110
BMI (kg/m2)	2.78	2.13	1.31	−1.39–6.95	0.194
Triglycerides (mmol/L)	0.84	1.44	0.59	−1.98–3.66	0.560
Urine pH	0.76	10.65	0.07	−20.11–21.63	0.943
GLP-1 use	−20.08	14.82	−1.36	−49.12–8.95	0.178
SGLT2 inhibitor use	−5.06	14.79	−0.34	−34.04–23.92	0.733
Baseline ACR (mg/g)	−0.00	0.02	−0.28	−0.04–0.03	0.781
Baseline uric acid	0.58	0.08	7.25	0.42–0.74	<0.001
Baseline GFR (mL/min)	0.53	0.46	1.14	−0.38–1.44	0.256
Finerenone dose					
−10 mg (reference)	—	—	—	0.00 (reference)	—
−20 mg	−7.40	14.06	−0.53	−34.96–20.15	0.599

The multivariable model (Table 5) demonstrated that baseline serum uric acid was the strongest independent predictor of ΔSUA (β = 0.58 per 1 μmol/L increase, p < 0.001). HbA1c also showed a weak positive association (β = 7.48, p = 0.040). The model found no significant difference in the magnitude of SUA reduction when comparing the 20 mg dose to the 10 mg reference dose (β = −7.40, p = 0.599). Similarly, SGLT2 inhibitor use was not an independent predictor of ΔSUA in this adjusted model (β = −5.06, p = 0.733). Consistently, in a linear mixed model additionally adjusted for SGLT2i and GLP-1 use, the time effect of finerenone on SUA reduction remained significant (β = −47.8, 95% CI: −63.4 to −32.2, p < 0.001), and neither concomitant medication was a significant independent predictor.

### 4.5 Safety

Finerenone was generally well-tolerated. Treatment-emergent hyperkalemia (serum potassium ≥5.5 mmol/L) was observed in one patient (0.8%). The condition resolved after temporary drug discontinuation and dietary adjustment, and the patient was successfully re-challenged with finerenone at 10 mg daily without recurrence.

## 5 Discussion

This real-world, retrospective study observed that finerenone treatment was associated with significant reductions in both albuminuria and serum uric acid (SUA) in patients with type 2 diabetic kidney disease (T2DKD). The observed SUA reduction averaged 47.9 μmol/L (≈12% from baseline). These findings suggest a potential dual metabolic and renal association, particularly relevant for DKD patients with concurrent hyperuricemia (Heerspink et al., 2020).

### 5.1 Interpretation of findings in context of existing evidence

The reduction in SUA observed in our cohort differs from findings in other studies. We propose that this discrepancy is primarily attributable to the distinct characteristics of our cohort, notably the high prevalence and severity of baseline hyperuricemia (Inker et al., 2012; Jin et al., 2023; Johnson et al., 2003). The mean baseline SUA in our cohort (400.0 μmol/L) was substantially higher than typically reported in RCT populations (Kolkhof and Bärfacker, 2017). This explanation is strongly supported by our subgroup analysis, which showed that the significant SUA reduction was almost entirely driven by patients with baseline hyperuricemia (mean decrease of 88.6 μmol/L). This indicates that any potential urate-modulating effect of finerenone may be most discernible in individuals with pre-existing elevated SUA (Kolkhof et al., 2015). Alternative explanations for the observed reduction must be acknowledged, including regression to the mean—a phenomenon likely in this short-term study of patients selected for high baseline SUA—and the absence of a control group. The small but significant eGFR decline (−2.7 mL/min/1.73 m^2^) is consistent with the acute hemodynamic effect expected with MRAs and mirrors findings from RCTs. (Levey et al., 2006; Liu et al., 2022). That this decline coincided with, rather than attenuated, the SUA reduction hints at a mechanism potentially independent of pure glomerular hemodynamics, possibly involving improved tubular handling of urate secondary to reduced renal inflammation.

### 5.2 Independence from concomitant therapies

A key ancillary finding is that the magnitude of SUA reduction appeared independent of concomitant use of SGLT2 inhibitors or GLP-1 receptor agonists (Liu et al., 2015). Subgroup and interaction analyses in both linear mixed and multivariable regression models found no significant effect modification or independent predictive value for these agents (Liu, 2024). This suggests that the observed association between finerenone and lower SUA is not merely a confounded effect of these commonly co-prescribed, urate-friendly medications. However, the possibility of residual confounding or unmeasured synergistic effects cannot be excluded.

### 5.3 Mechanistic considerations and clinical implications

The primary mechanism of finerenone—blocking mineralocorticoid receptor-driven inflammation and fibrosis—is well-established and aligns with the robust 39.4% reduction in UACR we observed (Naj et al., 2022; Navaneethan et al., 2025). We hypothesize that this attenuation of renal inflammation may secondarily improve the local environment for uric acid excretion, a pathway supported by preclinical links between inflammation and tubular urate transport (Perakakis et al., 2023; Perkovic et al., 2019). Regarding clinical relevance, it is important to note that this study was not designed to and cannot establish whether the observed short-term SUA reduction translates into additional long-term renal or cardiovascular protection beyond finerenone’s proven benefits. Our findings are hypothesis-generating (Pitt et al., 2021). They highlight a subgroup of patients—those with DKD and hyperuricemia—in whom finerenone might offer a particularly compelling therapeutic profile, potentially addressing two interrelated risk factors simultaneously (Sánchez-Lozada et al., 2005; Tentolouris et al., 2025). Future prospective studies are needed to confirm the durability of this effect and its impact on hard endpoints.

### 5.4 Limitations

Our study has important limitations that necessitate cautious interpretation. First and foremost, the lack of a control group precludes definitive causal attribution of the SUA reduction to finerenone; observed changes could reflect regression to the mean or other unmeasured temporal factors (Vaduganathan et al., 2025). Second, the retrospective, single-center design may introduce selection bias and limits generalizability. Third, the short follow-up (1–3 months) only captures initial biochemical responses; long-term effects on SUA dynamics remain unknown (Wada et al., 2025; Wang et al., 2025). Fourth, we lacked data on important lifestyle confounders (e.g., diet, alcohol intake) and biomarkers of inflammation or urate transport, which limits mechanistic insight (Zhu et al., 2011). Finally, the sample size, while adequate for detecting the main effect, provides limited power for some subgroup and multivariable analyses. Similar limitations have also been reported in other real-world studies on finerenone for diabetic kidney disease (Zhang et al., 2025).

## 6 Conclusion

In conclusion, this real-world analysis observed that finerenone use was associated with significant reductions in albuminuria and serum uric acid in patients with T2DKD, with the latter effect most pronounced in those with baseline hyperuricemia and appearing independent of SGLT2i or GLP-1RA use. These exploratory findings identify hyperuricemic DKD patients as a population of interest for future research into the potential pleiotropic metabolic effects of finerenone. Prospective, controlled studies with longer follow-up are warranted to confirm these associations and explore their clinical significance.

## Data Availability

The datasets presented in this article are not readily available because The dataset is available upon reasonable request for academic or research purposes. Interested researchers may contact the corresponding author via email to request access. The dataset is subject to the following restrictions: Usage Restriction: The data may only be used for non-commercial, academic, or scientific research purposes. Confidentiality: Users must agree not to attempt to re-identify any individuals from the de-identified data. Redistribution Prohibition: The dataset may not be shared, distributed, or published in its raw form without prior written permission from the authors. Attribution Requirement: Any publications or presentations resulting from the use of this dataset must appropriately cite the original source publication. If these conditions are met, the raw data can be provided via email upon request. Requests to access the datasets should be directed to linyamm54@163.com.
