# Supplementary figures and images for: Finerenone is associated with pronounced uric acid reduction in hyperuricemic diabetic kidney disease: a real-world analysis
Source: Front Pharmacol. 2026 Mar 2;17:1782658. doi: 10.3389/fphar.2026.1782658 (PMC12989612; doi:10.3389/fphar.2026.1782658)

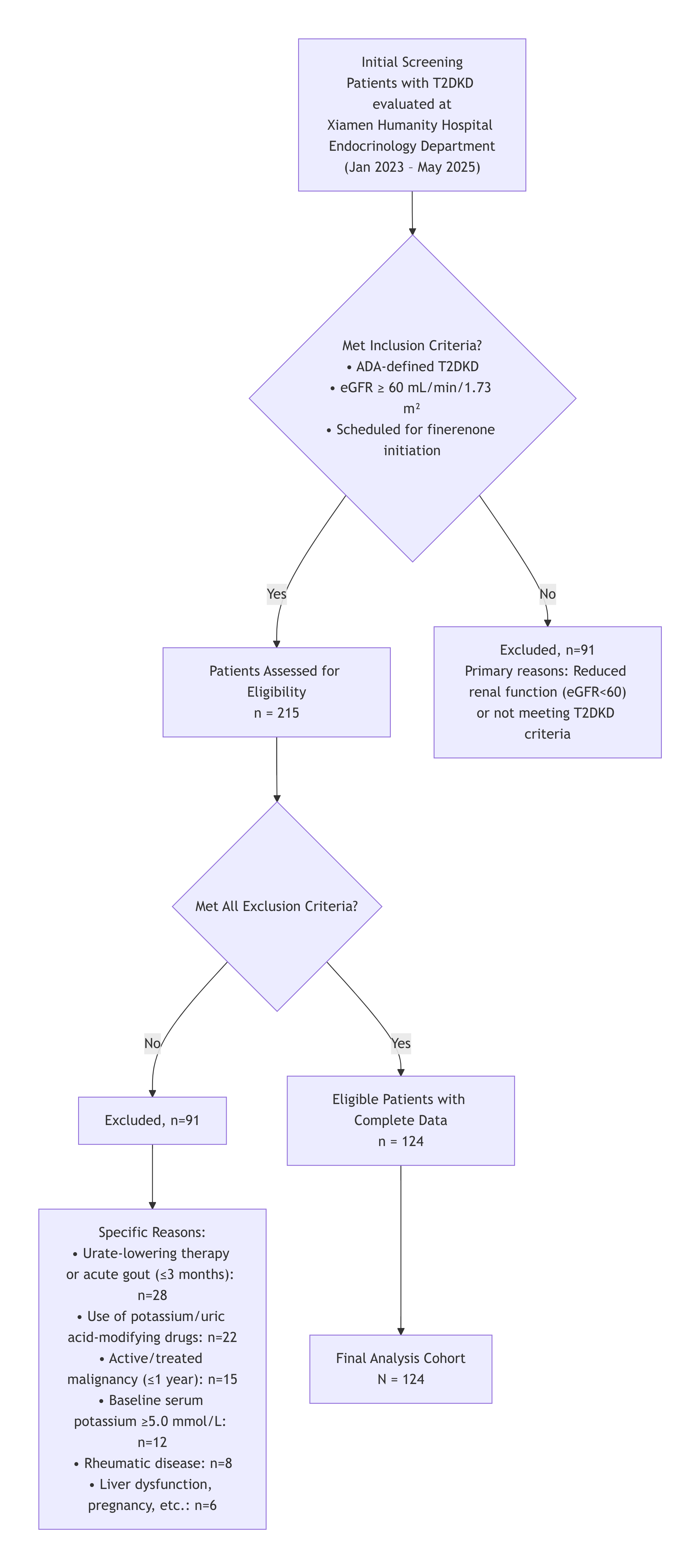

Supplement: Supplementary file 1 [file Image1.jpg]
